# Navigating Adult-Onset IgA Vasculitis-Associated Nephritis

**DOI:** 10.3390/life14080930

**Published:** 2024-07-25

**Authors:** Ming Ying Gan, Freda Zhi Yun Chua, Zi Yun Chang, Yan Ting Chua, Gek Cher Chan

**Affiliations:** 1 Department of Medicine, National University Hospital, Singapore 119074, Singapore; 2 Division of Nephrology, Department of Medicine, National University Hospital, Singapore 119074, Singapore; 3 National University Centre for Organ Transplantation, National University Hospital, Singapore 119074, Singapore; 4 Department of Medicine, Yong Loo Lin School of Medicine, National University of Singapore, Singapore 117597, Singapore

**Keywords:** IgA vasculitis (IgAV), IgA vasculitis nephritis (IgAVN), Henoch–Schonlein purpura

## Abstract

Purpose of Review: IgA vasculitis (IgAV), formerly Henoch–Schonlein purpura, is the most common systemic vasculitis in childhood. In adults, however, this condition is poorly understood, yet associated with more severe disease and poorer outcomes. This necessitates the need for early diagnosis and management. Scope of Review: We describe the pathophysiology, clinical manifestations, and diagnosis of IgAV in adults. Poor outcomes are often due to the high frequency of glomerulonephritis in IgAV-IgA vasculitis-associated nephritis (IgAVN). We hence also aim to summarize the latest clinical data regarding treatment strategies in IgAVN. The diagnosis and differentiation in histology between IgAVN and IgA nephropathy (IgAN) remain a challenge. Review of treatment therapies: Pathological mechanisms between IgAVN and IgAN appear to be consistent between the two, and data from IgAN are often extrapolated to IgAVN. The role of various immunosuppression therapies remains controversial, and in this review, we will discuss immunosuppression use and highlight evidence surrounding emerging and promising novel therapies in IgAVN/IgAN. Our aim for this review is to guide future treatment strategies and direct future studies.

## 1. Introduction

Immunoglobulin A vasculitis (IgAV) is a condition formerly known as Henoch–Schonlein purpura. It is an immune complex-mediated, small-vessel vasculitis which can involve the joints, kidneys, gastrointestinal tract, and skin. The 2012 Chapel Hill Conference [1] defined it as vasculitis with IgA1-dominant immune deposits, affecting small vessels in the skin and gastrointestinal tract and frequently causing arthritis. When this condition affects the kidneys, it is specifically termed Ig A vasculitis-associated nephritis (IgAVN) [2]. IgAVN is often indistinguishable from IgA nephropathy (IgAN), which presents more often in adulthood, and usually with macroscopic hematuria during an upper respiratory or gastrointestinal illness [3]. While the two conditions have a common pathological feature of significant mesangial IgA deposition, it remains controversial whether they could be two clinical manifestations of the same disease occurring on a spectrum, but varying in symptomatic presentations and prognosis [4].

IgAV is the most common systemic vasculitis in childhood, with an annual incidence of 3–26.7 per 100,000 children and a mean age of onset at 6 years [5]. In comparison, it is less common in adults, with an annual incidence of 0.1–1.8 per 100,000 individuals and a median age at onset of 50 years [5]. Hence, most of the existing literature stems from the pediatric population. IgAV is rare and it is poorly understood in adults, but it may be more prevalent than initially thought because of under-diagnosis [6]. Hence, patients may present and receive treatment at a later stage [7]. Therefore, IgAV in adults is also associated with more severe disease, necessitating a greater focus and need for management [6]. IgAV in adults has a more severe course and poorer outcome due to the high frequency of glomerulonephritis (IgAVN), which is the most serious complication of this vasculitis [8]. Preventing the development of established kidney inflammation is a priority to improve long-term outcomes. However, there is currently limited information about the histopathologic disease severity as related to disease onset [8]. Therapies are being developed based on the evolved understanding of the pathophysiology of IgAVN, which spans mucosal immunity, kidney inflammation, and complement pathway activation [9]. In recent years, there have been progressively more data on potential benefits of steroids, immunosuppressive therapies, and immune system targets that bring more clarity to possible novel therapies for this complex disease. 

Herein, we review the pathogenesis, clinical manifestations, and diagnosis of adult-onset IgAV. We also aim to summarize the current treatment therapies as well as potential immune targets in the therapeutic pipeline to direct future studies and offer recommendations for potential future studies specifically in IgAVN and IgAN.

## 2. Pathophysiological Mechanisms of IgAV 

The pathogenesis of IgAV is complex and involves multiple players, including both innate and acquired immunity, galactose-deficient IgA1 (Gd-IgA1) immunocomplexes, and environmental and genetic factors. A distinctive feature observed in IgAV is IgA1-dominant IgA deposits in small vessel walls. While IgAN and IgAVN differ in clinical features, prognosis, and outcomes, they seem to share similar underlying pathological mechanisms [10], which is based on a current concept often known as a “four-hit” hypothesis [11].

Immunoglobulin A (IgA) can be classified based on its location of production (serum or secretory) and subclasses (IgA1 and IgA2). IgA comprises two light chains and two heavy chains with a hinge region of amino acids [12]. In the hinge region of IgA1, there are three to six O-linked glycan sites where a process called O-glycosylation takes place. This process, through glycosidic linkages, involves the attachment of galactose (Gal) and N-acetylgalactosamine (GalNAc) with or without silic acid (Neu5ac) to oxygen atoms of serine/threonine residues [13]. O-glycosylation involves three important enzymes, namely, polypeptide *N*-acetylgalactosaminyltransferase 2 (GALNT2), core 1 β1,3-galactosyltransferase (C1GALT1), and sialyltransferases (α2,3-sialyltransferase for Gal and α2,6-GalNAc-sialyltransferase 2 for GalAc) [14,15,16,17,18,19,20].

**Figure 1:**
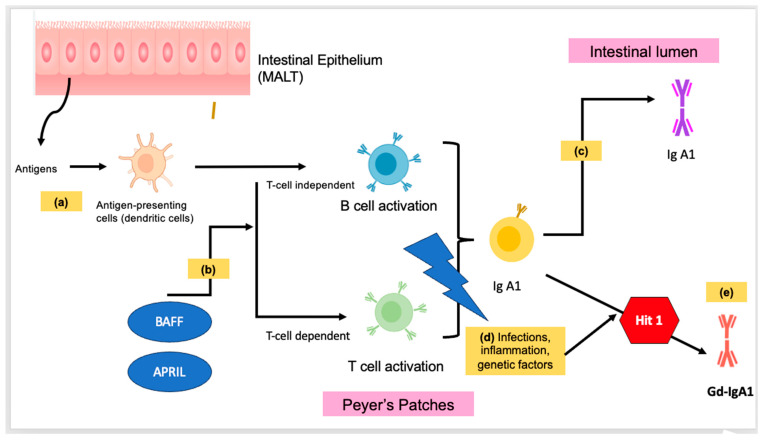
Illustration of the pathophysiological mechanisms of IgAV. (a) Within the mucosal-associated lymphoid tissue (MALT), antigens are taken up by antigen-presenting cells, such as dendritic cells. (b) Through dendritic cells, B-cell activation factor (BAFF) and a proliferation-inducing ligand (APRIL) can secrete cytokines that induce B cells to undergo class switching to IgA1. This requires a T-cell-independent or T-cell-dependent co-stimulatory signal. (c) In normal mucosal immunity, IgA-secreting plasma cells migrate to mucosa and release IgA1 into the lumen. (d) Infections, inflammation, and genetic factors can affect peripheral or mucosa-associated lymphoid tissue (MALT) involved in the activation of B cells. (e) Triggering of the mucosal immune response results in an abnormal glycosylation process, and hence galactose-deficient IgA1 (Gd-IgA1). This is also part of the first hit mechanism in the four “hit” mechanisms of IgAV.

Cytokines and gene polymorphisms play a role in the regulation of the above three enzymes. The production of mucosal IgA may be induced by T-cell-dependent or T-cell-independent mechanisms. The secretion of cytokines such as interleukin-6, interleukin-10, B-cell activation factor (BAFF) of the tumor necrosis family (TNF), and a proliferation-inducing ligand (APRIL) by dendritic cells induces B cells to undergo class-switching recombination from IgM to IgA1. IgA-secreting plasma cells then migrate to the mucosal lamina propria and release IgA1 into the lumen [21]. Figure 1 illustrates the pathophysiological mechanisms of IgAV. 

### 2.1. Increased Synthesis of Gd-IgA1 

**Figure 2:**
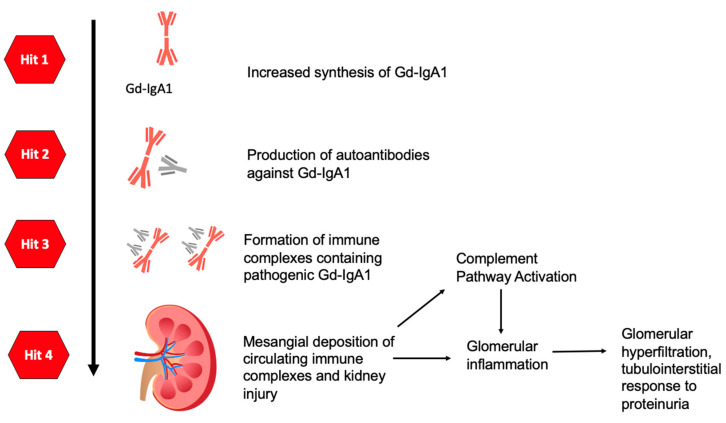
Diagram summarizing the four “hit” mechanisms of IgA. Hit 1: Production and increased synthesis of Gd-IgA1. Hit 2: Production of autoantibodies against Gd-IgA1. Hit 3: Formation of immune complexes containing pathogenic Gd-IgA1. Hit 4: Deposition of immune complexes in the kidneys, resulting in complement pathway activation as well as glomerular inflammation.

Gd-IgA1 is a recognized pathogenic abnormality in IgAN, where aberrant glycosylation of human IgA1 results in a deficiency of galactose in the hinge region of the IgA1 heavy chains [22]. This process was hypothesized to be facilitated by genetic predisposition and/or mucosal infection. It has been suggested that exposure to respiratory infections triggers a mucosal immune response, resulting in elevated production of Gd-IgA1 by peripheral or mucosa-associated lymphoid tissue (MALT, e.g., the tonsils or Peyer’s patches), which activates B cells, followed by the onset of IgAV or IgAN [1,9]. The formation of Gd-IgA1 forms part of the “first hit” mechanism of the pathophysiology of IgAV (Figure 2). 

### 2.2. Production of Autoantibodies against Gd-IgA1

Consequently, residues in Gd-IgA1 or mucosal antigens that mimic the structure of Gd-IgA1 lead to the upregulation and presentation of autoantibodies [23]. Studies have shown that IgG antibodies recognize the galactose-deficient hinge-region O-glycans of Gd-IgA1, and aberrantly glycosylated IgA1 is found nearly exclusively within immune complexes bound to IgG or IgA1 antibodies. Serum levels of IgG antibodies correlate with Gd-IgA1 levels, and these define an autoimmune component of IgAN [10,22]. 

### 2.3. Gd-IgA1 Immune Complex Formation

When circulating Gd-IgA1 is recognized by autoantibodies of IgA1 or IgG antibodies, it results in the formation of circulating immune complexes. It has been demonstrated that elevated Gd-IgA1 levels alone do not cause IgAV nor mesangial cell proliferation [24], but instead, the onset is triggered by the Gd-IgA1 immune complexes [25,26]. This emphasizes the pathological importance of Gd-IgA1 immune complex formation, through the binding of autoantibodies to Gd-IgA1 [23]. 

### 2.4. Immune Complex Deposition 

Following its formation, Gd-IgA1 immune complexes are deposited at the small vessel walls of either the skin or the mesangial cells of the kidney. There is a lack of correlation between serum Gd-IgA1 levels with the intensity of Gd-IgA1 deposits, suggesting that other factors may affect the deposition of these immune complexes [27]. 

A group of IgA1 receptors, transferrin receptors (TfRs) [28], are expressed in renal mesangial cells. Binding of Gd-IgA1 immune complexes to TfRs activates mesangial proliferation in IgAVN [28]. This in turn induces a proinflammatory cascade involving the release of cytokines and chemokine expression, and podocytes and tubular epithelial cells undergo apoptosis, which further enhances inflammatory injury in the kidneys [10,29,30,31,32]. The effects of Gd-IgA1 in IgAV patients without nephritis remain controversial. 

Figure 2 demonstrates the four “hit” mechanisms of IgAV, which consequentially lead to glomerular inflammation and IgAVN or IgAN. 

### 2.5. Role of Complement System 

**Figure 3:**
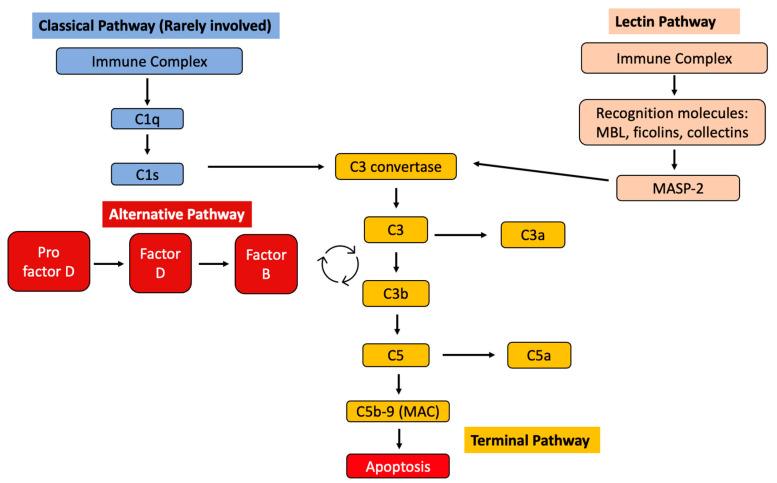
Diagram summarizing the involvement of complement pathways in IgAV. MBL = mannose-binding lectin; MASP-2 = mannose-binding lectin-associated serine protease.

Tissue deposition of Gd-IgA1 immune complexes can trigger local complement activation, but this process is not well understood [21]. The three main complement system pathways include the classical pathway (CP), the lectin pathway (LP), and the alternative pathway (AP), which converge in the terminal pathway (TP). The activation of LP, AP, and TP as effector mechanisms of kidney injury in IgAN has been demonstrated [21]. The absence of C1q in most IgAN kidney biopsies suggests that CP is not significantly involved in its pathogenesis. Figure 3 demonstrates the involvement of complement pathways in IgAV. 

The activation of complements is involved in tissue injuries in patients with IgAV. The activation of the alternative pathway is evident by the presence of elevated C3a, C5a, C4, and C5-9 deposits. Deposits of C4d and C5b-9 are associated with poor renal outcomes [33]. 

There has also been evidence of mannose-binding lectin (MBL) pathway activation reported in IgAV [34]. The activated complements induce upregulated expression of cytokines and recruit inflammatory cells [23]. 

### 2.6. Infections

Observations of IgAV onset following infections suggest that infections may also play a role in the pathogenesis of IgAV. Several hypotheses have been proposed. First, pathogens’ surfaces may contain immunoglobulins that recognize Gd-IgA1, facilitating the production of cross-reactive immunoglobulins. Second, microorganisms can contain antigenic structures resembling those of vessel walls that induce the production of cross-reactive autoantibodies. Lastly, mucosal infection can upregulate IL-6, altering the glycosylation machinery and producing Gd-IgA1 [23]. Associations between various pathogens with IgAV have been described, and these include Helicobacter Pylori, Staphylococcus, Streptococcus, Parvovirus, and Hepatitis B virus [35]. 

### 2.7. Genetics

Genetic factors may be implicated in the pathogenesis of IgAV, although there have been no proven mutations with direct causation. Significant geographic and ethnic differences in the prevalence of this disease suggest a possible genetic role [36]. Asians show a relatively higher incidence of IgAV than Caucasians, while Africans, Caribbeans, and the Indian subcontinent Asian population exhibit the lowest incidence rate of this pathology [36]. There is also increased IgAV risk described among first-degree relatives of affected patients as well as familial aggregation [36], suggesting that genetic factors underlie the pathogenesis of this disorder. 

Certain HLA genes have also been associated with IgAV, with HLA-B*41:02 found to be a susceptibility marker for IgAV development [23]. HLA genes code for MHC molecules, which present antigens to T cells. T-cell activation can affect autoimmunity and hence could potentially implicate IgAV susceptibility. 

## 3. Clinical Features and Associations of IgAV

The classical presentation of IgAV includes cutaneous manifestations and gastrointestinal, joint, and renal involvement. These manifestations typically develop over days or weeks and may vary from individual to individual, between children and adults [37]. 

### 3.1. Cutaneous [38]

Symmetrical palpable purpura with edema is almost always present [37]. It is usually located in the dependent areas such as the lower extremities, and it may extend to the trunk and upper extremities [39]. In about one-third of the cases, it may become necrotic or hemorrhagic, and cutaneous exacerbations may be seen for 6 months or longer [40,41]. 

### 3.2. Gastrointestinal 

Gastrointestinal (GI) manifestations are common, occurring in two-thirds of IgAV patients, and their incidence is similar between children and adults. GI symptoms are due to submucosal hemorrhage, wall edema, and vasculitis of small and large intestines [39]. They vary in severity, ranging from colicky abdominal pain, nausea, vomiting, melena, and/or rectorrhagia to serious manifestations including intussusception, infarction, necrosis, and bowel perforation [42]. Other rare hepatobiliary manifestations include acute primary biliary cirrhosis, peritonitis, elevated liver enzymes, pancreatitis, and acalculous cholecystitis [43].

### 3.3. Joint 

Arthralgias are also common and occur in two-thirds of IgAV cases [44]. They are usually transient and non-deforming in nature, and can involve multiple joints (mainly the knees and ankles) [37]. Frank arthritis with joint effusion is uncommon. Occasionally, myalgia has been reported. 

### 3.4. Renal 

Proteinuria, microscopic or macroscopic hematuria, and/or the presence of red blood cell casts take place in approximately 76.2% of IgAV patients [35]. Microscopic or macroscopic hematuria with or without red cell casts is the earliest and most sensitive sign suggestive of IgAVN [37]. The incidence of acute kidney injury can reach up to 32% in adults at time of diagnosis but is rare in children [38]. In the long term, 10% to 30% of patients with IgAVN develop end-stage renal disease at 15 years of follow-up [45,46,47]. Clinical factors such as hypertension and level of proteinuria are predictors of poor renal outcome in adult-onset IgAVN [38,46].

An observational cohort found that elderly patients with IgAV were more likely to present with end-stage renal disease, while young adults had outcomes similar to childhood-onset IgAV [48]. 

### 3.5. Others

Pulmonary manifestations [49] such as alveolar hemorrhage occur more in adults than in the pediatric population but are extremely rare. IgAV can involve other organs and present with (i) cardiac manifestations such as myocarditis, cardiac arrhythmias, and valvulitis [50]; (ii) ophthalmological manifestations (episcleritis, uveitis, scleritis, and keratitis); and (iii) central and peripheral nervous system manifestations (peripheral neuropathy, cerebral vasculitis, and posterior reversible encephalopathy [51]).

### 3.6. Clinical Associations

IgAV in adults can be associated with infections, malignancies [52], autoimmune conditions, and pathogens as elaborated on above, such as Helicobacter Pylori, Staphylococcus, Streptococcus, Parvovirus, and Hepatitis B virus [35]. 

There is also an association between IgAV and malignancy—predominantly solid tumors such as lung cancer [53], followed by prostate and hematological cancers [54]. Hence, age- and gender-appropriate malignancy screening should be performed in adult-onset IgAV [55]. 

## 4. Diagnosis 

Three IgAV diagnostic criteria have been developed for children. They are (i) the 1990 American College of Rheumatology (ACR) diagnostic criteria, (ii) the revised 2012 Chapel Hill International Consensus Conference for Nomenclature of Vasculitides, and (iii) the 2010 European League against Rheumatism/Pediatric Rheumatology International Trials Organization/Pediatric Rheumatology European Society (EULAR/PRINTO/PRES) diagnostic criteria (Table 1). Among them, due to the lack of validated diagnostic criteria in adults, the 2010 EULAR/PRINTO/PRES criteria are often used in clinical practice due to their higher sensitivity and specificity scores (sensitivity of 99.2% and specificity of 86% compared to 86.8% sensitivity and 81% specificity of ACR criteria) [35].

In the absence of a specific diagnostic test, physicians have to rely on clinical features and histopathological findings to diagnose IgAV in adults. If IgAV is suspected in the presence of a rash, platelet count and coagulation studies should first be performed to exclude other causes of purpura. Histological confirmation with a skin biopsy is important in adults. Light microscopy usually reveals a classic leukocytoclastic vasculitis predominantly in postcapillary venules [58]. In fresh lesions (of less than 48 h), direct immunofluorescence examinations often demonstrate IgA with complement (C3) depositions. Even though IgA deposits are associated with IgAV, they are not pathognomonic as they can also be found in other forms of vasculitides. 

Renal function must be evaluated in adults. In addition to serum creatinine and estimated GFR, urinary studies should be performed to confirm the presence of hematuria, dysmorphic red blood cells, or red blood cell casts [35], and to quantify the amount of proteinuria. Serum IgA levels may be elevated, but they are not considered a diagnostic marker and do not have prognostic significance [59]. If there is suspected renal involvement, a kidney biopsy is recommended in adults with (i) renal function impairment, (ii) persistent proteinuria >1 g/day despite the use of renin–angiotensin–aldosterone system (RAAS) inhibitors, and (iii) nephrotic or nephritic syndrome, or rapidly progressive glomerulonephritis [35,55]. 

## 5. Histological Findings and Relevance on Prognosis 

IgAVN often has overlapping features with IgAN, which is the most common glomerulonephritis worldwide [60]. Both are characterized by hematuria and proteinuria, and they share a common pathogenic basis with glomerular mesangium deposition of immune complexes, but a reliable way to differentiate IgAN from IgAVN histologically is lacking [35]. One observation study found that renal impairment at biopsy was an independent risk factor for the subsequent development of ESKD [48].

Others have suggested that histology in IgAV with nephritis shows more capillary staining and glomerular injury than in IgAN [60]. It is important to differentiate between the two disease entities for prognostication purposes, and distinguishing IgAV often relies on the clinical presentation of extra-renal manifestations. IgAN has a poorer outcome than IgAVN, with 30–40% of patients reaching end-stage renal disease 20–30 years after first clinical presentation [61]. In comparison, a study found that in IgAVN, 31.6% of adults may have renal functional impairment, with end-stage renal disease observed in 15.8% of adults [45]. 

A review of the literature shows that renal involvement in IgAV occurs in about 33% of children and 63% of adults. The most typical manifestation is segmentary focal glomerulonephritis, often associated with granular IgA deposits in the mesangium [61]. Other findings include classic leukocytoclastic vasculitis prominently in postcapillary venules on light microscopy, granular IgA-dominant or -co-dominant deposits within glomeruli on immunofluorescence, and the presence of electron-dense deposits in the mesangial areas on electron microscopy [35]. 

Different histologic classifications have been proposed for IgAN as well as IgAVN. Efforts have also been undertaken to characterize the prognostic implications of the renal histopathologic lesions on IgAVN [62].

(A) International Study of Kidney Disease in Children (ISKDC)

The International Study of Kidney Disease in Children (ISKDC) Classification, which is used for the classification of IgAVN, describes basic morphological changes based on crescents and categorizes renal biopsy results into one of six histological grades with the first five based on presence and number of crescents and Grade VI representing membranoproliferative-like glomerulonephritis characterized with changes in the mesangial elements and glomerular capillary wall. The classification is based mostly on the state of glomeruli but mainly reflects active inflammation, neglecting vascular and tubulointerstitial changes [63]. This classification relies mainly on the presence of crescents and is relevant only for children [62].

(B) Oxford Classification

Secondly, the Oxford Classification used in IgAN was also utilized to include changes to sclerotic glomeruli and interstitial fibrosis, which correlates better with long-term outcome and could be used to predict the progression of renal disease in IgAVN [63]. This scoring system published in 2009 mainly included four morphological features: mesangial hypercellularity (M), endocapillary proliferation (E), segmental glomerulosclerosis/adhesion (S), and tubular atrophy/interstitial fibrosis (T). In a 2016 revision, an additional C (crescent) score was added—now widely used as the MEST-C scoring system. While the MEST-C scoring system provides prognostic information for those with IgAN, studies that attempted to use MEST-C to predict outcomes in IgAV have yielded mixed results [35]. This scoring system is not recommended for clinical use in IgAVN [35] as IgAVN patients were not included in the validation cohort [63]. 

As IgAVN and IgAN have similar pathological features that are indistinguishable, a recent study reviewed kidney biopsies from 262 children and 99 adults with IgAVN. The MEST-C scores were correlated with eGFR and degree of proteinuria at the time of biopsy, together with clinical risk factors. The study found that renal outcomes of IgAVN patients were determined by endocapillary hypercellularity (E1) lesions, which are not part of the ISKDC classification. Those who responded well to immunosuppression initially remained at risk of developing disease progression later, therefore necessitating the need for long-term follow-up [64]. This study utilized mixed class models to associate both histologic and clinical variables, a commonly used approach to evaluate eGFR trajectories in chronic kidney disease (CKD), allowing the identification of eGFR trajectory patterns in an unbiased manner [62].

(C) Haas Classification

The Haas Classification for IgAN is based on retrospective studies and uses the development of end-stage renal disease as the primary endpoint. This histologic classification demonstrated a statistically significant correlation between IgA subclass and renal survival [63].

(D) Semiquantitative Classification (SQC)

Another classification is the semiquantitative classification (SQC), developed by Koskela et al. in 2017 [63]. This classification considers various variables, evaluating both the activity and chronicity components. Glomerular, tubular, interstitial, and vascular findings are scored, and the maximum score is defined as the total biopsy score [63]. This research yielded promising results but did not have sufficient patient numbers for validation [63]. It was also mainly used in the pediatric population rather than the adult population. 

A recent study compared the above four classifications in the prediction of renal outcomes. The SQC proved to be the best in outcome prediction followed by the Oxford classification [65]. The Oxford parameters for mesangial hypercellularity and tubular atrophy, as well as SQC parameters for cellular crescents, showed an independent statistically significant contribution to outcome prediction [65]. Despite the above, there are no specific targeted histological classifications for IgAVN in adults. 

More research in this field is warranted to predict disease outcomes and prognosis. 

## 6. Treatment of IgAVN 

Herein, we shall focus on the management of adult-onset IgAVN. The treatment of extra-renal manifestations and pediatric-onset IgAV are beyond the scope of this review. 

In those without rapidly progressing glomerulonephritis (RPGN), the KDIGO 2021 Guidelines recommend that IgAVN patients should receive supportive care. It is a combination of cardiovascular risk assessment, lifestyle advice, and modifications such as smoking cessation, weight control, regular exercise, and blood pressure control to a standardized office measurement of systolic blood pressure of <120 mmHg. Titration of RAAS inhibitors to a maximal tolerated dose without immunosuppressive therapy should be utilized [66]. IgAVN patients with persistent proteinuria of >1 g/day despite optimized supportive care for at least 90 days are at high risk of progressive CKD. 

The evidence in adult-onset IgAVN remains limited, and much is often extrapolated from IgAN. High-risk patients in whom immunosuppression is considered, especially in those with an eGFR < 50 mL/min/1.73 m^2^, should undergo individually tailored counseling on the risks and benefits of immunosuppressive therapy. A six-month tapering course of glucocorticoid therapy, of which evidence was derived from IgAN, may be considered [67,68], and should be given with extreme caution or avoided entirely in those with contraindications [67]. 

Based on the KDIGO 2021 Guidelines, beyond glucocorticoids, other immunosuppressive therapies are not recommended in IgAN, including Azathioprine, Cyclophosphamide (except in the setting of rapidly progressive IgAN), calcineurin inhibitors (CNIs), and Rituximab [67]. The use of Mycophenolate mofetil (MMF) in IgAN is not recommended in non-Chinese patients, whereas it may be used as a glucocorticoid-sparing agent in Chinese patients [67]. Similarly, in non-Japanese patients, there are no data to support the routine use of tonsillectomy in high-risk IgAN patients. If immunosuppression is being considered, a detailed discussion of the risks and benefits of each drug should be undertaken with the patient, recognizing that adverse treatment effects are more likely in patients with an eGFR < 50 mL/min per 1.73 m^2^.

### 6.1. Glucocorticoids

Corticosteroids, intravenous or oral, are part of most treatment regimens, and there is some evidence of their positive result on the long-term outcome of adult IgAN patients [69]. STOP-IgAN (The Supportive versus Immunosuppressive Therapy for the Treatment of Progressive IgAN) trial was a multicenter, open-label, randomized controlled trial (RCT) that compared glucocorticoids to supportive therapy. Patients who had persistent proteinuria with urinary protein excretion of at least 0.75 g per day were randomly assigned to receive supportive care alone or supportive care with immunosuppressive therapy for 3 years [70]. The study demonstrated that the addition of immunosuppression did not provide any benefit to renal function; instead, more adverse effects were observed among the patients who had received immunosuppressive therapy, with no change in the rate of decrease in the eGFR [70]. 

On the contrary, the TESTING randomized controlled trial demonstrated some benefits of glucocorticoids on renal outcomes. They initially randomized IgAN patients, with proteinuria greater than or equal to 1 g/day, to 6 to 9 months of oral Methylprednisolone at 0.6–0.8 mg/kg/day. However, the study was prematurely terminated due to serious infections at higher-dose Methylprednisolone, prompting a dose reduction by tapering oral Methylprednisolone at 0.4 mg/kg/day (maximum 32 mg/day) over 6 to 9 months, with the addition of antibiotic prophylaxis for pneumocystis pneumonia. The results showed that among patients with IgAN at high risk of progression, treatment with lower-dose oral Methylprednisolone significantly reduced the risk of outcome of kidney function decline, kidney failure, or death due to kidney disease [71].

As per KDIGO recommendation, given the risk of serious adverse events, an individualized risk assessment must be conducted and a tailored course of glucocorticoids after a detailed risk–benefit discussion between the managing physician and patient [68]. 

### 6.2. Mycophenolate Mofetil (MMF)

MMF exerts a reversible inhibitory effect on T- and B-lymphocyte proliferation by inhibiting type I and II inosine monophosphate dehydrogenase, which prevents de novo guanosine nucleotide synthesis and DNA synthesis [72]. There are several studies demonstrating the benefits of MMF in patients with IgAVN and IgAN. 

There were two retrospective studies from China on patients with IgAVN. First, Ren et al. reviewed 53 patients with biopsy-proven IgAVN with proteinuria > 2 g/day. The age of patients ranged from 14 to 62 years, and the median age was 27 years. It compared a group of MMF (1.0–1.5 g/day) combined with low-dose Prednisone (0.4–0.5 mg/kg/day) with the full-dose Prednisone group (0.8–1.0 mg/kg/day). The overall remission rates were similar between both groups, while the MMF group had a lower relapse rate [73]. 

Second, another retrospective study reviewed 95 patients who fulfilled the EULAR/PRINTO/PRES-endorsed consensus criteria for the classification of childhood vasculitides and had persistent proteinuria 1.0 to 3.5 g/day after at least three months of RAAS inhibition, an eGFR based on the Modification of Diet in Renal Disease (MDRD) equation >60 mL/min/1.73 m^2^, and a follow-up period of at least 6 months [74]. They were divided into three groups of therapy after at least three months of RAAS inhibition. The MMF group received MMF 1.0–1.5 g/day combined with tapering Prednisone (0.4–0.5 mg/kg/day); the corticosteroid group received tapering Prednisone 0.8–1.0 mg/kg/day, while the control group continued with RAAS inhibitors at an optimized dose. Patients in the MMF and corticosteroid groups continued to take RAAS inhibitors at the original dose. All groups were treated with a year of therapy. The mean range of age across the three groups was 29 to 35 years. The study showed proteinuria reduction and better remission rates in both the MMF and corticosteroid groups, compared to the control group. However, the corticosteroid group had the highest number of adverse events, followed by the MMF group. 

A meta-analysis reviewing eight studies proposed that patients with IgAN treated with MMF had higher remission than the control group [69]. Another randomized trial, conducted in China, compared 170 adults with IgAN who received either MMF plus supportive care or supportive care alone. The study showed that the addition of MMF to supportive care reduced the risk of disease progression among patients with progressive IgAN [75]. 

MMF may be considered as a glucocorticoid-sparing agent in Chinese patients, but it is not recommended in non-Chinese patients [67]. The long-term outcomes of MMF use in IgAV are not well described in the existing literature.

### 6.3. Hydroxychloroquine (HCQ)

Hydroxychloroquine is an anti-malarial drug with immunomodulatory and anti-inflammatory effects. It has been widely used for the treatment of autoimmune diseases, such as systemic lupus erythematosus and rheumatoid arthritis. Potential mechanisms for HCQ in IgAN or IgAVN include interference with immune activation at various cellular levels by inhibiting the innate and adaptive immune systems, which acts to stimulate the production of galactose-deficient IgA1 [76]. 

A systematic review of five studies described the HCQ effect on proteinuria in patients with IgAN. Based on the study, HCQ treatment in addition to optimized supportive therapy significantly and safely reduced proteinuria [76], but did not demonstrate benefits on estimated glomerular filtration rate (eGFR). However, there has only been one retrospective case–control study demonstrating that HCQ reduces proteinuria in Chinese patients with IgAVN [77]. 

### 6.4. Azathioprine

Current data available suggest that Azathioprine is ineffective and may even be toxic in IgAN [78]. An early retrospective analysis of 74 patients followed for 10 years showed that long-term Azathioprine with low-dose Prednisolone did not alter the clinical course compared to untreated controls. A more recent prospective randomized study of 207 subjects showed that the addition of Azathioprine to corticosteroids did not confer additional health benefits in terms of kidney survival [78]. It is not a recommended therapeutic agent in the management of IgAN [68]. The evidence of Azathioprine use is also limited to pediatric-onset IgAVN. This is based on an observational trial that showed that 60% of patients who received the combination of Azathioprine and glucocorticoids achieved clinical remission [79]. 

### 6.5. Cyclophosphamide 

Cyclophosphamide is an alkylating agent. It decreases DNA synthesis and crosslinks DNA strands to prevent cell division, and it is used in various vasculitides. The evidence on the use of Cyclophosphamide in adults with IgAV remains controversial. The KDIGO guidelines recommend the use of Cyclophosphamide and glucocorticoids in patients with rapidly progressive IgAN, which is defined as a 50% or more decline in eGFR over ≤3 months (where other etiologies have been excluded), based on data derived from other glomerular diseases and the lack of other therapeutic options available to rescue this high-risk cohort from end-stage kidney disease [80]. A Cochrane review in 2020 did not demonstrate a protective effect of CYC with regard to end-stage kidney disease or complete remission [81]. 

However, in the setting of IgAV with RPGN, the KDIGO guidelines recommend an individual risk assessment and a risk–benefit discussion with the patient and treatment in accordance with guidelines for ANCA-associated vasculitis. In addition, it is crucial to elicit extra-renal manifestations that warrant other immunosuppressive strategies [68]. There have been two trials (one prospective and one retrospective) comparing the use of glucocorticoids alone with the use of glucocorticoids and Cyclophosphamide in patients with adult-onset IgAV. Both studies demonstrated no difference between both groups in remission rates [82,83]. 

### 6.6. Calcineurin Inhibitors (CNIs)

Calcineurin inhibitors (CNIs), such as Cyclosporin and Tacrolimus, inhibit T-cell activation. There is very limited weak evidence on the use of calcineurin inhibitors in adult-onset IgAV/IgAVN, and it largely demonstrates a reduction in proteinuria, while the long-term outcomes remain unknown. A meta-analysis conducted in 2017 [84] showed that CNIs might provide renal protection in patients with IgAN, but at an increased risk of adverse events. There is a need for more high-quality trials with large sample sizes to reliably evaluate the efficacy and safety of CNIs in IgAN. Current guidelines do not recommend the use of CNIs in IgAN due to limited evidence [68].

(A) Cyclosporin

Cyclosporin has known regulatory effects on the immune system and hence was evaluated for usefulness in the treatment of IgAN. A randomized prospective single-blind study of 19 patients in 1989 with IgAN and proteinuria noted a significant reduction in proteinuria with treatment with Cyclosporin [85]. A case series of five patients with adult-onset IgAVN and nephrotic-range proteinuria received a combination of Cyclosporin and glucocorticoids. It demonstrated the effectiveness of this combination therapy in proteinuria reduction while maintaining stable renal function at five years of follow-up [86].

(B) Tacrolimus

Tacrolimus use in IgAV is limited to pediatric cohorts in whom off-label use of Tacrolimus was evaluated, and it showed significant proteinuria reduction after six months [87]. A prospective cohort study assigned 50 patients with biopsy-proven IgAN to receive oral Tacrolimus or full-dose glucocorticoids for 6 months [88]. It showed that Tacrolimus was non-inferior to full-dose glucocorticoids in inducing proteinuria remission at 6 months, suggesting an alternative option of Tacrolimus in patients who are unwilling to use full-dose glucocorticoids. However, the long-term impact on renal function needs to be monitored [88].

A double-blind trial studied the antiproteinuric effect of Tacrolimus for 40 biopsy-proven IgA nephropathies for 16 weeks [89]. Patients were randomly assigned either to receive Tacrolimus or placebo with an RAAS inhibitor. It revealed a greater reduction in the mean value of urinary albumin–creatinine ratio at 12-week and 16-week visits in the Tacrolimus group compared to the control group. Tacrolimus effectively reduced proteinuria in normotensive patients with IgAN. This suggested that Tacrolimus could be an alternative to corticosteroids and RAAS inhibitors for IgAN patients who cannot tolerate anti-hypertensive medication [89]. 

## 7. Novel Therapies of IgAN 

In the 2021 Kidney Disease Improving Global Outcomes (KDIGO) guidelines for glomerular diseases, it was acknowledged that no specific therapies for IgAN were available [66]. The historical lack of drug development in IgAN was largely attributable to kidney outcomes that may be ‘difficult’ to achieve and a lack of clinical trials [66]. However, regulatory authorities now accept proteinuria reduction as a reasonable surrogate endpoint for progression to kidney failure. This hence accelerated the approval of new treatments in IgAN, transforming the IgAN clinical trial landscape [66]. With these new clinical trial activities, there is more hope for the treatment of IgAN and IgAVN. 

Pathological mechanisms such as abnormal O-glycosylation IgA1 immune complex creation, glomerular deposition, involvement of complement cascade, and T-cell-independent mechanisms for mucosal B-cell activation appear to be similar between IgAN and IgAV. Hence, drugs that target these processes being trialed in IgAN may also potentially benefit patients with IgAVN [9]. 

**Figure 4:**
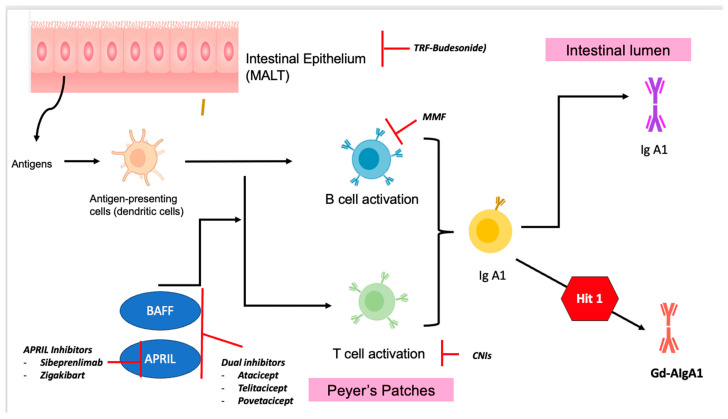
Diagram illustrating the targeted therapies of IgAN based on the pathophysiology of IgAN. MMF = Mycophenolate Mofetil; CNIs = calcineurin inhibitors; TRF-Budenoside = Targeted-Release Formulation Budenoside; APRIL inhibitors = a proliferation-inducing ligand (APRIL) inhibitors; BAFF inhibitors = B-cell activation factor (BAFF) inhibitors.

**Figure 5:**
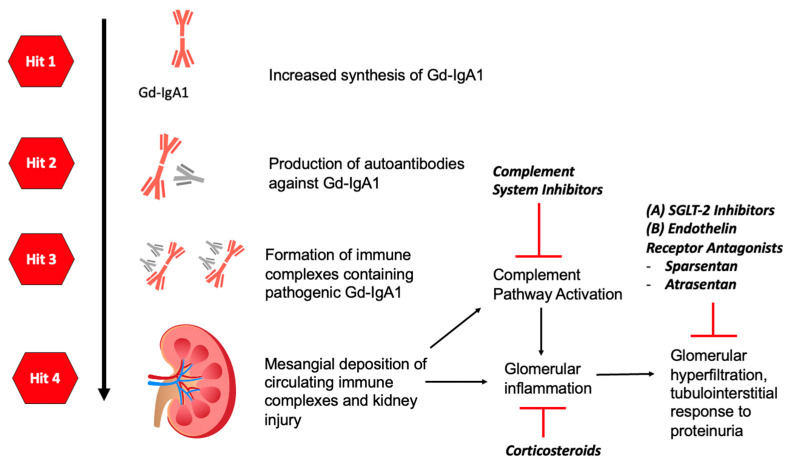
Diagram illustrating the “four hit” mechanisms in IgAN and targeted therapies. SGLT-2 inhibitors = sodium–glucose cotransporter-2 inhibitors.

**Figure 6:**
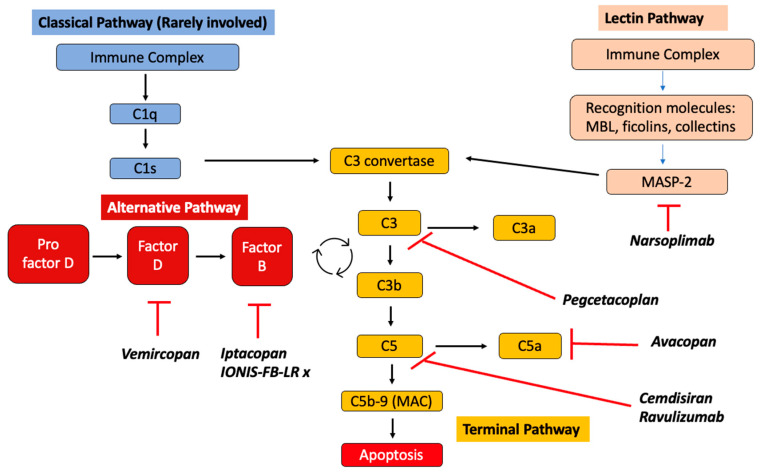
Diagram illustrating the complement activation pathways, and sites of targeted therapies. MBL = mannose-binding lectin; MASP-2 = mannose-binding lectin-associated serine protease.

Understanding the pathophysiology of IgAN allows us to better understand sites of targeted therapies in the treatment of IgAN. These are illustrated in Figure 4, Figure 5 and Figure 6. Herein is a summary of novel therapies of IgAN thus far, summarized based on pathophysiological mechanisms. Unfortunately, most of these trials have excluded patients with IgAV. A summary of the latest novel therapies and their associated trials in IgAN have also been consolidated in Table 2. 

### 7.1. Targets of Gut Immune System (against Formation of Gd-IgA1) 

The gut mucosal immune system and mucosal-derived Gd-IgA1 are thought to be involved in the pathogenesis of IgAN. Peyer’s patches are collections of lymphoid follicles found in the mucosal layer of the intestine and concentrated in the ileum [101]. They belong to the gut-associated lymphoid system, functioning as antigen-sampling and inductive sites and a source of mucosal B cells that express Gd-IgA1. As we have alluded to, Gd-IgA1 plays an important role in the pathogenesis of IgAN. Hence, drugs targeting the gut-associated lymphoid system may reduce Gd-IgA1 production by inhibiting mucosal B-lymphocyte activation and Peyer plaque proliferation [101]. 

### 7.2. TRF-Budesonide

The Targeted-Release Formulation (TRF) of Budesonide, Nefecon, is an oral formulation of Budesonide. It is designed to deploy in the ileum and deliver the drug locally to the ileal gut-associated lymphoid system, where Peyer plaque density is the highest, thus limiting systemic steroid exposure [102,103]. The NeflgArd study was a randomized controlled phase 3 trial to evaluate the efficacy, safety, and tolerability of Nefecon 16 mg/day in adult patients with primary IgAN at risk for progression to kidney failure [102]. After a 9-month period of treatment, patients receiving Nefecon achieved a reduction in eGFR decline and proteinuria at 24 months as compared to placebo [102]. 

### 7.3. B-Cell-Directed Therapy

B cells play a key role in the pathogenesis of IgAN as the source of Gd-IgA1. B-cell-activating factor (BAFF) and a proliferation-inducing ligand (APRIL), members of the tumor necrosis factor superfamily, regulate both T-cell-dependent and T-cell-independent class-switch recombination (CSR) [104]. The pathogenesis of IgAN is tied to aberrations in B-cell activation and CSR that lead to increased production of Gd-IgA1 (Hit 1), which further leads to Hits 2–4 in the pathogenesis pathway. Given their pivotal roles in the production of Gd-IgA1 and their autoantibodies, targeting BAFF and APRIL offers a logical therapeutic strategy for IgAN [104].

(A) A Proliferation-Inducing Ligand (APRIL)-Neutralizing Monoclonal Antibodies [100]

There are two ongoing phase 3 studies evaluating the use of humanized monoclonal antibodies that inhibit APRIL in patients with IgAN—Sibeprenlimab (VISIONARY Trial, NCT05248646) and Zigakibart (BEYOND Trial, NCT0502911). 

Sibeprenlimab is a humanized IgG2 monoclonal antibody that inhibits APRIL. The phase II randomized controlled trial ENVISION has shown greater reductions in proteinuria from baseline compared to placebo [105]. Zigakibart is another novel humanized anti-APRIL monoclonal antibody. Interim results from a phase 1/2 trial of Zigakibart in patients with IgAN (NCT03945318) demonstrated rapid and durable reductions in Gd-IgA1, along with sustained proteinuria reductions and an acceptable safety profile [106].

(B) Dual antagonists of BAFF and APRIL

Fusion proteins contain extracellular portions of T-cell activator and calcium-modulating ligand binder that can bind and inhibit both BAFF and APRIL, therefore downregulating and preventing downstream activation of signaling pathways associated with B-cell homeostasis. There are three agents undergoing evaluation: Atacicept (NCT04716231), Telitacicept (NCT05799287), and Povetacicept (in progress).

A phase 2b ORIGIN trial randomized 116 individuals with biopsy-proven IgAN to varying doses of once-weekly Atacicept versus placebo, for up to 36 weeks [92]. The results showed that at week 24, there was a reduction in the mean urine protein creatinine ratio from baseline by 25% in the Atacicept group versus placebo, and a 35% reduction at week 36. This was also accompanied by a stabilization in endpoint eGFR and Gd-IgA1 levels [92]. There is an ongoing phase 3 RCT comparing Atacicept 150 mg once-weekly subcutaneous injections with placebo in patients with IgAN (ORIGIN 3—NCT04716231) [93]. 

Telitacicept was also shown to reduce proteinuria in patients with IgAN in a phase II randomized controlled trial [107]. Similarly, a phase 3 RCT comparing Telitacicept 240 mg once-weekly subcutaneous injections with placebo in patients with IgAN is ongoing (NCT05799287).

Povetacicept was evaluated in a phase 1b/2a RUBY-3 study of biopsy-proven autoimmune glomerulonephritis including IgAN. Forty-one patients with IgAN received Povetacicept 80 mg or 240 mg. Treatment at both doses was associated with improvements in proteinuria, stable renal function, and reductions in Gd-IgA1 [95].

### 7.4. B-Cell-Depleting Agents

Rituximab is an anti-CD20 monoclonal antibody that depletes peripheral B cells. At present, the use and efficacy of this medication in patients with IgAVN are still limited. There is no demonstrated benefit in proteinuria reduction, kidney function, or reduction in the levels of Gg-IgA1. Several case reports have recommended that it could be effective in IgAVN in both pediatric and adult groups, especially if other lines of immunosuppressive treatments have failed to induce remission [69]. However, a randomized controlled trial of CD20 depletion in IgAN failed to demonstrate the benefit of Rituximab on top of standard therapy in proteinuria reduction, stabilization of renal function, or reduction in Gd-IgA1 and anti-Gd-IgA1 antibody levels [100]. 

Felzartamab is an anti-CD38 therapy currently evaluated in early-phase studies in IgAN. Felzartamab has shown efficacy in the preliminary phase in anti-phospholipase A2 receptor (PLA2R) antibody-positive MN. The proteasome inhibitor Bortezomib (Velcade) is also being studied in a pilot open-label trial involving eight patients with IgAN. More trials are needed to demonstrate efficacy and safety [100]. 

### 7.5. Non-Immunologic Therapy

(A) Sodium–Glucose Cotransporter-2 Inhibitors (SGLT2is)

Persistent proteinuria of >1 g/day is a predictor of renal function decline and increased mortality in patients with IgAN [108]. 

SGLT2is inhibit the reabsorption of sodium and glucose via SGLT2 channels in the proximal tubule. This increases delivery of sodium, chloride, and water to the macula densa, leading to tubuloglomerular feedback. Subsequently, this reduces intraglomerular perfusion pressure and overall filtration, contributing to a decrease in urinary protein excretion. There have been two trials evaluating SGLT2is in patients with IgAN, namely DAPA-CKD (Dapagliflozin) and EMPA-KIDNEY (Empagliflozin).

In the DAPA-CKD trial, Dapagliflozin attenuated albuminuria by 26% and significantly reduced the risk of major adverse kidney events by 71% in IgAN compared to the placebo group [108]. The EMPA-KIDNEY Trial included 817 patients with IgAN, and the primary composite outcome of the progression of kidney disease or death from cardiovascular cause occurred less frequently in the Empagliflozin group compared to placebo [100]. Combining results from EMPA-KIDNEY and DAPA-CKD showed a 51% reduction in risk of CKD progression in IgAN [109]. Another study by Dong et al. also demonstrated a reduction in proteinuria of 22.9% and 27.1% relative to the baseline after three and six months of SGLT2i treatment, respectively [108]. 

Thus far, there are minimal published trials that demonstrate the efficacy of SGLT2 in treating non-diabetic kidney disease. However, there are ongoing RCTs underway to focus on SGLT2is in reducing proteinuria in non-diabetic patients, such as the ADAPT Trial (NCT04794517) [110].

(B) Endothelin Receptor Antagonist

Endothelin-1 (ET-1) activates endothelin A (ETA) receptors across multiple kidney cell types, and this can lead to vasoconstriction, podocyte injury, inflammation, and fibrosis, which leads to the progression of CKD [100,111], which in turn contributes to IgAN pathogenesis. 

Sparsentan is a non-immunosuppressive dual endothelin and angiotensin receptor antagonist with high selectivity for endothelin receptor type A (ETaR) and angiotensin II receptor type 1 [111]. The PROTECT trial is a double-blind, randomized, active-controlled, phase 3 study which randomly assigned patients to receive 400 mg oral Sparsentan once daily or 300 mg oral Irbesartan once daily with the primary endpoint of proteinuria change. Interim analysis showed that the Sparsentan group had a significant and greater reduction in proteinuria from baseline (~49.8%) versus Irbesartan (−15.1%) at 36 weeks. Treatment-emergent adverse events with Sparsentan were similar to those with Irbesartan. It was granted accelerated approval by the US Food and Drug Administration in 2023 for the treatment of patients with IgA nephropathy at high risk of progression [111]. The final analysis of Sparsentan at more than 110 weeks of treatment showed that proteinuria, as defined as the change from baseline in urine protein-to-creatinine ratio, was 40% lower in the Sparsentan group as compared to Irbesartan [111]. 

Atrasentan is another potent and selective ETA antagonist which has been shown to reduce proteinuria and preserve kidney function in patients with IgAN who are at high risk of progression. AFFINITY is a phase 2 study that evaluated the efficacy and safety of Atrasentan in patients with proteinuric glomerular diseases [97]. It demonstrated that the treatment of patients with IgAN with Atrasentan in addition to standard of care provided a >43% reduction in proteinuria after 12 weeks and was well tolerated [97]. ALIGN is a randomized, multicenter, double-blind, placebo-controlled phase 3 trial that is comparing the efficacy and safety of 0.75 mg oral Atrasentan once daily to placebo, which is still ongoing (NCT04573478).

The ASSIST study (NCT05834738) [112], a phase 2 randomized, double-blind, placebo-controlled crossover study evaluating the safety and efficacy of Atrasentan versus placebo in patients with IgAN while on standard-of-care therapy as well as SGLT2is, is also underway [112]. This study is estimated to be completed in 2026. 

### 7.6. Complement Pathway Inhibitor

The complement system is an important component of innate immunity and can be activated through the aforementioned pathways described in the pathogenesis section. Complement activation via the LP, AP, and TP is the effector mechanism that underlies kidney injury in IgAN [21]. The absence of C1q in most IgAN kidney biopsies suggests that CP is not involved in its pathogenesis [21]. These pathways have become potential therapeutic options for the treatment of IgAN.

(A) Lectin Pathway Inhibition—Inhibition of MASP-2

The lectin pathway (LP) is activated by the binding of pattern recognition molecules to pathogen-associated molecular patterns. Pattern recognition molecules include mannose-binding lectin (MBL), ficolins, or collections which work with MBL-associated serine protease 1 (MASP-1) and 2 (MASP-2) [113]. 

MASP-2 is a component of the lectin pathway that triggers C3 convertase formation. Drug therapies targeting the inhibition of MASP-2 have been evaluated. Narsoplimab is a monoclonal antibody that selectively inhibits MASP-2, and it is administered via weekly infusions. A phase II trial demonstrated that the Narsoplimab group achieved proteinuria reduction at 18 weeks, suggesting that it was safe and well tolerated [114]. However, ARTEMIS-IgAN, a double-blind, randomized, placebo-controlled study that aimed to evaluate the safety and efficacy of Narsoplimab, was terminated when interim analysis did not demonstrate a significant proteinuria reduction with Narsoplimab [100] compared to placebo.

(B) Alternative Pathway Inhibition

The alternative pathway (AP) is the main activator of the complement cascade in IgAN, and glomerular C3 deposits correlate with progression of disease. Factor B is a crucial cofactor for C3 activation and AP activity, where a positive correlation has been observed between plasma levels of Factor B and levels of Gd-IgA1. The mechanism of IgA-mediated alternative pathway activation remains poorly understood, but it is hypothesized to require stabilization of the C3 convertase [115]. As the alternative complement pathway includes two key proteins—Factor B and D—drugs inhibiting these two proteins are being evaluated. 

Iptacopan is a potent oral selective inhibitor of Factor B. A phase 3 trial (APPLAUSE-IgAN) evaluating the efficacy and safety of Iptacopan is currently ongoing, but a prespecified interim analysis demonstrated the superiority of Iptacopan over placebo in proteinuria reduction at 9 months [22]. 

Another antisense inhibitor of complement Factor B production is RO7434656 (IONIS-FB-LRx). Its efficacy and safety profile are being evaluated in an ongoing phase 3, multicenter, randomized, double-blind, placebo-controlled study (IMAGINATION) in patients with primary IgA nephropathy (NCT05797610). 

Vemircopan, a Factor D inhibitor, is also currently being studied in a phase II study in patients with IgAN and proliferative lupus nephritis (NCT05097989) [100].

(C) Terminal Pathway Inhibition

The terminal pathway (TP) also appears to be important in the pathogenesis of IgAN. The generation of C5b triggers the terminal sequence that culminates in the formation of C5b-9, and this deposition is associated with kidney inflammation and progression of glomerulosclerosis. Mesangial deposits of this terminal pathway complete complex, also called membrane attack complex, are commonly observed in IgAN [115]. 

C3 and C3b inhibitors such as Pegcetacoplan have been evaluated in an open-label, phase 2 study on their preliminary efficacy and safety for various complement-mediated glomerular diseases including IgAN [99]. It has found that there was a reduction in mean proteinuria, suggesting possible therapeutic benefit [99].

Ravulizumab is a monoclonal antibody against C5 also being evaluated in a phase II clinical trial in patients with proliferative lupus nephritis or IgAN (SANCTUARY—NCT04564339). Preliminary analysis showed improvements in proteinuria reduction compared to placebo at 26 weeks (40.3% vs. 10.9%) [100]. This analysis suggests the clinically meaningful efficacy of Ravulizumab based on rapid and sustained proteinuria reduction, providing proof of concept for a phase 3 trial as a potential treatment of IgAN [116]. 

Cemdisiran is a small interfering RNA that blocks terminal complement pathway activation and consequent inflammation by suppressing C5 production in the liver. Results from a phase II study comparing subcutaneous Cemdisiran 600 mg or placebo every 4 weeks in combination with the standard of care showed a 37.4% mean reduction in proteinuria and stabilization of GFR in favor of Cemdisiran at week 32 [100]. The mean change in serum C5 levels from baseline at week 32 was −98.7% with Cemdisiran and 25.2% with placebo [117]. 

C5a is a strong chemoattractant that recruits immune cells into sites of inflammation. Avacopan is a selective C5a receptor antagonist that can dampen the complement-mediated inflammatory response, which has shown benefit in the treatment of anti-neutrophil cytoplasmic antibody-associated vasculitis (ANCA vasculitis). A pilot open-label phase II trial of Avacopan has shown improvement in proteinuria in six out of seven patients during the treatment period, with three having a numerical improvement of proteinuria of about 50%. More trials are needed to confirm the efficacy and safety of C5a inhibition in IgAN [118]. 

## 8. Treatment and Research Recommendations in IgAVN 

In this paper, most of the findings presented were in relation to IgAN, including treatment therapies and trials, as there is currently still limited evidence on the management of adult-onset IgAVN. Most therapeutic trials available on IgAN excluded patients with IgAVN and as such, it is not yet possible to conclude their effectiveness in this cohort. Whilst some say that IgAN and IgAVN have similar pathological features and treatment strategies can be extrapolated, some have also argued that they are exclusive diseases with their own treatment guidelines. As adult-onset IgAVN is a rare diagnosis, there are currently insufficient data to compare treatment efficacies. More research studies ought to be conducted to determine the clinical outcomes of these novel therapies among the IgAVN adult population. 

There have been individual case series or case reports describing positive outcomes in adults with IgAVN who have been treated with B-cell-depleting therapies [119], or with other immunosuppressants such as MMF and Cyclophosphamide [120]. While these case series may demonstrate the effectiveness of therapy in patients with IgAVN, repeated treatments may be necessary and further follow-ups may be needed to evaluate long-term clinical outcomes and the safety of treatment [119]. However, the evidence for these therapeutic options remains weak. 

In the meantime, KDIGO guidelines have highlighted that in the absence of sufficient long-term data, IgAVN should be treated in the same way as in patients with non-severe forms of isolated IgAN. However, it is notable that the KDIGO guidelines do not take into account the more acute onset of IgAVN with more aggressive lesions in renal histology [67]. 

## 9. Conclusions

This review comprehensively updates the most recent knowledge and understanding of IgAV. The understanding of the pathophysiology of IgAVN as well as IgAN is important in the development of therapeutic agents directly targeting mechanistic pathways. With improved understanding of the pathogenesis, more therapeutic agents have emerged, giving hope to this complex and rare disease which conventionally lacks specific therapies. There is a myriad of potential drugs that could be used as early targets to prevent progression warranting further evaluation. It is important to recognize the current limited evidence on the management of adult-onset IgAVN, and even though much data have been extrapolated from IgAN, more trials and studies are needed to validate its effectiveness in IgAVN patients. This is also a significant limitation of this paper, where many of the findings presented in this article were for IgAN and extrapolated to IgAVN. 

In addition to treatment guidelines, establishing an accurate and early diagnosis, as well as prognosticating IgAV, is also important for disease monitoring and treatment. More studies are warranted to identify and validate various kidney biomarkers to better inform prognostication, treatment selection, and methods of monitoring response to treatment. 

## Figures and Tables

**Table 1:** ACR, Chapel Hill, and EULAR/PRINTO/PRES diagnostic criteria.

Classification Title	Diagnostic Criteria
ACR diagnostic criteria (1990) [56]	Two of the following: - Age < 20 years old; - Palpable purpura; - Acute abdominal pain; - Biopsy showing granulocytes in the walls of small arterioles or venules.
1994 Chapel Hill Criteria [35] (Revised in 2012)	IgAV was defined as vasculitis with IgA1-dominant immune deposits, affecting small vessels (predominantly capillaries, venules, or arterioles) that often involve the skin and gastrointestinal tract, and cause arthritis and glomerulonephritis.
EULAR/PRINTO/PRES diagnostic criteria (2010) [57]	Purpura or petechiae and one of the following: - Abdominal pain; - Arthritis or arthralgia; - Renal involvement; - Leukocytoclastic vasculitis with predominant IgA deposits, proliferative glomerulonephritis, and predominant IgA deposits.

**Table 2:** Summary of latest novel therapies and their associated trials in IgAN.

Sites of Therapy Based on Pathophysiology	Therapy	Trials	Inclusion Criteria	Outcomes
Gut mucosal immune system	Targeted-Release Budesonide—Nefecon	NEFIGAN phase 2b	>18 years old, with biopsy-confirmed primary IgAN, persistent proteinuria > 0.5 g/day despite optimized RAAS inhibitors, eGFR > 45 mL/min/1.73 m^2^ 150 patients randomized into 3 groups: placebo, half-dose Budesonide at 8 mg/day, or full-dose Budesonide at 16 mg/day (all still on RAAS inhibitors) for 9 months [90]	9-month treatment with Nefecon resulted in reduced proteinuria and stabilized eGFR
		NefigArd Phase 3	≥18 years old, with primary IgAN, eGFR 35–90 mL/min per 1.73 m^2^, and uPCR ≥ 0.8 g/g or proteinuria ≥ 1 g/24 h) despite optimized RAAS inhibitors 364 patients randomized into 2 groups: 16 mg/day oral Nefecon and matching placebo for 9 months [91]	9-month treatment with Nefecon provided a clinically relevant reduction in eGFR decline and a durable reduction in proteinuria versus placebo
APRIL-neutralizing monoclonal antibodies	Sibeprenlimab	RCT Phase 3 trial—NCT05248646	Phase 2 multicenter double-blind randomized controlled trial evaluated 12 monthly intravenous infusions of Sibeprenlimab at doses of 2, 4, or 8 mg per kilogram body weight versus placebo Phase 3 trial >18 years old, biopsy-confirmed IgAN, stable and maximally tolerated dose of RAAS inhibitors for at least 3 months, screening uPCR ≥ 0.75 g/g or urine protein ≥ 1.0 g/day, eGFR ≥ 30 mL/min/1.73 m^2^ 530 patients randomized into 2 groups: Sibeprenlimab 400 mg subcutaneously every 4 weeks compared to placebo for 9 months	Phase 2 trial demonstrated greater reduction in 24 h uPCR from baseline as compared to placebo Ongoing phase 3 trial
	Zigakibart	NCT05852938 BEYOND study	Ongoing phase 3 trial >18 years old, biopsy-proven IgAN, eGFR ≥ 30 mL/min/1.73 m^2^, total urine protein ≥ 1.0 g/day and uPCR ≥ 0.7 g/g as measured from an adequate 24 h urine collection, stable on a maximally tolerated dose of ACEi/ARB for at least 12 weeks, body mass index (BMI) between 18 and 40 kg/m^2^, screening weight of 45 to 150 kg. 292 patients randomized into 2 groups: Zigakibart 600 mg SC every 2 weeks versus placebo for total of 104 weeks	Reduction in proteinuria as early as 12 weeks with an associated reduction in Gd-IgA1 levels
Antibody inhibiting APRIL and BAFF	Atacicept	ORIGIN Trial [92] (NCT04716231) ORIGIN III Trial [93]	Double-blind, placebo-controlled phase 2b clinical trial with biopsy-proven IgAN 116 patients randomized in a 2:2:1:2 fashion to Atacicept 150 mg, 75 mg, 25 mg versus placebo Ongoing phase 3 trial ≥18 years old, 24 h total urine protein excretion ≥ 1.0 g or uPCR ≥ 1.0 mg/mg based on a 24 h urine sample during the screening period, biopsy-proven IgAN, eGFR > 30 mL/min/1.73 m^2^, on stable prescribed RAAS inhibitors, SBP < 150 200 patients randomized into 2 groups: Atacicept 150 mg versus placebo	Phase 2b clinical trial showed a dose-dependent reduction in proteinuria and Gd-IgA1 antibody levels Phase 3 trial is ongoing
	Atacicept	JANUS Trial [94] (NCT02808429)	Phase 2 study >18 years old, biopsy-proven IgAN, screening uPCR > 0.75 to <6 mg/g, stable ACEi/ARB for at least 8 weeks 16 patients randomized in a 1:1:1 fashion to Atacicept 25 mg, 75 mg versus placebo	Reduction in proteinuria and Gd-IgA levels and is safe to be used in IgAN patients.
	Telitacicept	NCT04905212	Phase 2 multicenter, randomized, double-blind, controlled trial >18 years old, biopsy-proven IgAN, 24 h total protein ≥ 0.75 g, eGFR > 30 mL/min per 1.73 m^2^, on ACEi/ARB, diuretics, or other anti-hypertensives 44 Chinese patients randomized in 1:1:1 fashion to Telitacicept 160 mg, 240 mg versus placebo	Ongoing phase 3 trial
	Povetacicept	RUBY-3 Trial [95]	A multiple-ascending-dose, multi-cohort, open-label, phase 1b/2a study of Povetacicept ≥18 years of age, biopsy-confirmed autoimmune glomerulonephritis including IgAN, biopsy-confirmed diagnosis ≤ 10 years prior to the start of screening, screening uPCR ≥ 0.5 g, on maximal tolerated ACEi/ARB dose for at least 12 weeks. 41 patients who received either Povetacicept 80 mg or 240 mg.	Treatment with both doses was found to be associated with reduction in proteinuria, stable renal function, and reduction in Gd-IgA1 with good safety profile [95]
Depletion of plasma cells	Rituximab (anti-CD 20)	RCT NCT04525729	Phase 4 trial with Rituximab combined with RAAS inhibitors will be compared with RAAS inhibitors for IgAN patients, to explore a more effective and safer regimen for IgA >18–75 years old, biopsy-proven IgAN, eGFR > 30 mL/min/1.73 m^2^, 24 h proteinuria > 1 g with maximal tolerated ACEi/ARB for 3 months, serum albumin > 25 g/L 116 patients randomized to 2 groups: Rituximab with RAAS inhibitors versus RAAS inhibitors	Did not show reduction in proteinuria, stabilization of renal function, or reduction in Gd-IgA1 and anti-Gd-IgA1 antibody levels
		RCT [96]	An open-label, multicenter study conducted over 1-year follow-up Biopsy-proven IgA nephropathy and proteinuria > 1 g/d, maintained on ACEi/ARB with well-controlled BP and eGFR < 90 mL/min per 1.73 m^2^ 34 patients randomized into 2 groups: standard with Rituximab versus standard therapy	It did not significantly improve renal function or reduction in proteinuria
	Felzartamab (anti-CD 38)	RCT (NCT05065970)	Randomized, placebo-controlled, multicenter, double-blind, 2a trial ≥18 to ≤80 years old, biopsy-proven IgAN, proteinuria at screening visit ≥ 1.0 g/day, adequate treatment with ACEi/ARB for ≥3 and adequate blood pressure (BP) control.	Efficacy shown in preliminary phase 1/2A trials in anti-phospholipase A2 receptor (PLA2R) antibody-positive membranous nephropathy
	Bortezomib	RCT	Bortezomib has been studied in a pilot open-label trial involving 8 patients with IgAN	3 out of these 8 patients achieved complete remission after 4 doses of Bortezomib at 1-year follow-up, suggesting that plasma cell depletion could potentially improve outcomes in IgAN
Non-immunotherapy	SGLT-2	DAPA-CKD	eGFR > 25 and <75 mL/min/1.73 m^2^, uPCR > 200 mg/g and <5000 mg/g, on single-agent RAAS inhibitor for 4 weeks	Sustained decline in eGFR by 50%, ESKD or death from cardio-renal causes
		EMPA-KIDNEY	eGFR > 30, <45 mL/min/1.73 m^2^ OR eGFR > 45, <90 mL/min/1.73 m^2^ with uPCR > 300 mg/g	Sustained declined in eGFR by 40%, or to 10 ESKD or death from cardio-renal causes
		SGLT2 Inhibitor Meta-Analysis Cardio-Renal Trialists’ Consortium (SMART-C)	Meta-analysis of 13 randomized controlled trials to provide pooled estimates of effect of SGLT2is	SGLT2is reduced the risk of kidney disease progression by 40% in patients with glomerular disease, specifically in IgAN
		ADAPT Trial (NCT04794517)	Randomized, prospective, double-blind, placebo-controlled phase 2b 18 years old, non-diabetic CKD-IV, persistent proteinuria (24 h urinary protein excretion ≥ 0.5 g in at least two consecutive evaluations >1 week apart), eGFR 15 to 30 mL/min/1.73 m^2^, maximal tolerated ACEi/ARB 93 patients randomized into 2 groups: 10 mg Dapagliflozin and placebo group	Ongoing trial, in phase 2
Endothelin receptor antagonists	Sparsentan	PROTECT trial	A double-blind, randomized, active-controlled, phase 3 study 18 years old, biopsy-proven IgAN, proteinuria > 1 g/day, eGFR ≥ 30 mL/min/1.73 m^2^, maximally tolerated ACEi/ARB 203 patients randomized in 1:1 fashion: either Sparsentan 400 mg or Irbesartan 300 mg	Interim analysis showed a significant reduction from baseline in proteinuria (~49.8%) versus Irbesartan (−15.1%) at 36 weeks
		SPARTACUS trial NCT05856760	Phase 2 trial >18 years old, biopsy-proven IgAN, uACR ≥ 0.3 g/g at screening, An eGFR value of ≥25 mL/min/1.73 m^2^ at screening and stable use of SGLT2is 12 weeks prior to screening 60 patients randomized into 2 groups: receiving Sparsentan and SGLT2is	Ongoing trial
	Atrasentan	AFFINITY trial [97]	Open-label phase 2 trial >18 years old, biopsy-proven IgAN, maximally tolerated ACEi/ARB dose, uPCR between 0.5 and less than 1.0 g/g, screening eGFR ≥ 30 mL/min/1.73 m^2^ 20 patients received 0.75 mg Atrasentan daily	>43% reduction in proteinuria after 12 weeks
		Phase 3 trial—ALIGN [98]	Randomized, multicenter, double-blind, placebo-controlled phase 3 clinical trial Biopsy-proven IgAN, maximally tolerated ACEi/ARB dose for at least 12 weeks, screening 24 h total protein > 1 g/day, eGFR ≥ 30 mL/min/1.73 m^2^ 320 patients randomized into 2 groups: receiving 0.75 mg Atrasentan versus placebo daily for 132 weeks, with stable dose of SGLT2is	At week 36, Atrasentan achieved significant reduction in proteinuria compared to placebo
Complement pathway inhibitors	Lectin pathway—MASP2 inhibitor: Narsoplimab	ARTEMIS-IGAN	Randomized, double-blind, placebo-controlled trial phase 3 trial >18 years old, with biopsy-proven IgAN within 8 years of screening, screening 24 h total protein > 1 g/day, eGFR ≥ 30 mL/min/1.73 m^2^ 450 patients randomized into 2 groups in 1:1 fashion of weekly Narsoplimab versus placebo for 12 weeks	The ARTEMIS-IGAN trial did not show statistical reduction in proteinuria as compared to placebo Proteinuria reduction in the placebo group was substantially greater than reported in other IgA nephropathy clinical trials
	Alternative pathway—Factor B inhibitor: Iptacopan	APPLAUSE-IgAN	Multicenter, randomized, double-blind, placebo-controlled phase 3 trial ≥18 years old, with biopsy-proven primary IgAN at high risk of progression to kidney failure, maximally tolerated ACEi/ARB dose 470 patients randomized into 2 groups: Iptacopan 200 mg twice daily versus placebo for 24 months	Interim analysis demonstrated significant proteinuria reduction with Iptacopan as compared to placebo at 9 months
	Alternative pathway—Factor B inhibitor: IONIS-FB-LRX	NCT04014335	Single-arm open-label phase 2 trial >18–75 years old, females who are not pregnant and non-lactating, with biopsy-proven IgAN, with proteinuria and hematuria 25 patients, receiving IONIS-FB-LRx for 24 weeks	Ongoing trial
	Alternative pathway—Factor D inhibitor: Vemircopan	NCT05097989	Randomized, double-blind, placebo-controlled, multicenter phase 2 trial >18–≤75 years old, biopsy-proven lupus nephritis or IgAN, 24 h mean proteinuria ≥ 1 g/day on 2 occasions, stable dose of ACEi/ARB, well-controlled blood pressure 70 patients randomized into 3 groups: Vemircopan 120 mg and 180 mg and placebo for 24 weeks	Ongoing trial
	Terminal pathway inhibitor: Pegcetacoplan	NCT03453619 [99]	Open-label phase 2 trial >18 years old, with biopsy-proven IgAN, LN, primary MN, or C3G, proteinuria > 750 mg/g (either 24 h urine collection or uPCR), eGFR ≥ 30 mL/min/1.73 m^2^, stable or worsening renal disease despite on stable and optimized treatment 21 patients received Pegcetacoplan subcutaneous infusion daily for 16 weeks	Reduction in mean proteinuria from baseline to week 48 of 50.9% in the intent-to-treat population and 65.4% in the per-protocol population. eGFR was stable over 48 weeks. Mean soluble C5b-9 levels decreased by 57.3% by week 48.
	Terminal pathway—C5 inhibitor: Ravulizumab	NCT04564339	Double-blind, randomized, placebo-controlled phase 2 trial >18–75 years old, proteinuria ≥ 1 (g/day or g/g), vaccinated against meningococcus, Haemophilus influenzae type b (Hib), and Streptococcus pneumoniae, diagnosis of primary IgAN, compliance with stable and optimal RAAS treatment for ≥3 months 120 patients randomized into 4 groups: lupus nephritis receiving Ravulizumab, lupus nephritis with placebo, IgAN receiving Ravulizumab and IgAN with placebo for at least 26 weeks.	Reduction in proteinuria compared to placebo at 26 weeks (40.3% vs. 10.9%) [100]
	Terminal pathway—C5 inhibitor: Cemdisiran	NCT03841448	Randomized, double-blind, placebo-controlled phase 2 trial >18–65 years old, 24 h proteinuria > 1 g/day and hematuria, currently being treated for IgAN with stable, optimal therapy, including RAAS inhibitors. 31 patients randomized into 2 groups receiving Cemdisiran injection and placebo.	Ongoing trial
	Terminal pathway—C5a inhibitor: Avacopan	NCT02384317	Open-label, phase 2 trial Diagnosis of IgAN, eGFR > 60 mL/min/1.73 m^2^, proteinuria (first morning uPCR > 1 g/g)	Ongoing trial
